# Efficacy of tafamidis in transthyretin amyloid cardiomyopathy: a systematic review and meta-analysis

**DOI:** 10.1097/MS9.0000000000001482

**Published:** 2023-11-07

**Authors:** Mahnoor Sukaina, Shafi Rehman, Marium Waheed, Muhammad Shehryar, Raffat Rasool, Nimra Ahmed, Sidhant Ochani, Md. Al Hasibuzzaman

**Affiliations:** a Department of Medicine, Karachi Medical and Dental College, Karachi; b Department of Medicine, Khyber Medical College, Peshawar; c Department of Medicine, King Edward Medical University, Lahore; d Department of Medicine, Khairpur Medical College, Sindh, Pakistan; e Institute of Nutrition and Food Science, University of Dhaka, Dhaka, Bangladesh

**Keywords:** ATTR-CM, efficacy, meta-analysis, systematic review, tafamidis

## Abstract

In May 2019, the U.S. Food and Drug Administration approved tafamidis as the first conservative management of transthyretin amyloid cardiomyopathy (ATTR-CM). Our aim in conducting this systematic review and meta-analysis was to assess the efficacy of tafamidis on patients with ATTR-CM. For that purpose, we thoroughly searched PubMed, ScienceDirect, and Clinical trails.gov by using the appropriate search strategy and following predefined inclusion and exclusion criteria, which retrieved 235 articles initially. Of which two randomized controlled trials (RCTs) and one observational study matched our inclusion criteria. A total of 876 patients are included in this analysis. Based on results, tafamidis significantly reduced cardiovascular (CV) mortality in the ATTR-ACT trial and Ochi *et al*. (OR 0.58; 95% CI: [0.41–0.83], *P*=0.003, *I* ^2^=87%). A subgroup analysis was conducted for CV mortality due to heart failure (OR 0.89; 95% CI: [0.63–1.25], *P*=0.50, *I* ^2^=93%). The results exhibit that tafamidis reduced all causes of mortality (OR 0.45; 95% CI: [0.32–0.64], *P*≤0.00001, *I* ^2^=22%). Furthermore, mortality remained statistically insignificant in patients with heart transplants (OR 1.18; 95% CI: [0.52–2.70], *P*=0.70, *I* ^2^=0%) and patients with cardiac mechanical assist devices (OR 4.15; 95% CI: [0.48–35.66], *P*=0.20, *I* ^2^=0%). This meta-analysis suggests that tafamidis is a safe and efficient drug to use in patients with ATTR-CM and can possess the potential to be a milestone in enhancing the conservative management of the patients.

## Introduction

Highlights

- Transthyretin amyloid cardiomyopathy (ATTR-CM) is characterized by the deposition of amyloid fibrils, which are misfolded transthyretin proteins in the cardiac muscles.
- Tafamidis is a new promising treatment regimen that is a selective transthyretin stabilizer.
- This formal meta-analysis demonstrates that tafamidis is effective in reducing all causes of mortality and cardiovascular mortality in patients with ATTR-CM.

Transthyretin amyloid cardiomyopathy (ATTR-CM) is a fatal disease depicted by the accumulation of amyloid fibrils composed of misfolded transthyretin protein in the heart. Transthyretin is a 127-amino acid, a 55-kD protein synthesized and secreted by the liver in the plasma, and its function is to transport thyroxine and vitamin A^1^. Generally, transthyretin circulates in the blood as a tetramer. However, genetic mutations or aging cause dissociation of the tetramer into unstable monomers or oligomers that form insoluble amyloid fibrils, which accumulate and beget inimical effects on tissues and organ function, most commonly heart and nervous system^2^. There are two types of ATTR amyloidosis: ATTRvt, the hereditary form caused by pathogenic mutations of the transthyretin gene. And ATTRwt, previously called senile systemic amyloidosis, is caused by the deposition of wild-type transthyretin protein^1^. Until recently, there was no conservative management for ATTR-CM, Orthotopic Liver Transplantation (OLT) was carried out as the inaugural disease-modifying therapy^3^. In May 2019, FDA-approved tafamidis^4^ as the first conservative management of ATTR; it is a selective transthyretin stabilizer that prevents tetramer dissociation and amyloidogenesis^5^. To the best of our knowledge, this is the first meta-analysis assessing the efficacy of tafamidis on patients with ATTR-CM vs. the control group. The rationale of this study is to highlight the efficacy of the drug tafamidis, which subsequently halts the disease progression and deposition of misfolded transthyretin protein in the myocardium. In contrast to the usual conservative treatment that only treats the symptomatology rather than preventing the causative agent from reaching the myocardium.

## Methods

Two independent authors, M.S. and M.W., initiated a search from 2 June 2022, till 10 June 2022, on PubMed, ScienceDirect, and Clinical trails.gov using the following search strategy (Tafamidis OR FX 1006A OR Vyndaqel) AND (“Transthyretin Amyloid Cardiomyopathy” OR “amyloid cardiomyopathy” OR “light-chain amyloidosis” OR “TTR amyloid cardiomyopathy”). The work has been reported in line with AMSTAR (Supplemental Digital Content 1, http://links.lww.com/MS9/A294) (Assessing the methodological quality of systematic reviews) Guidelines (Supplemental Digital Content 2, http://links.lww.com/MS9/A295). The work has been reported in line with PRISMA (Supplemental Digital Content 3, http://links.lww.com/MS9/A296) (Preferred Reporting Items for Systematic Reviews and Meta-Analyses) Guidelines and is reported in PROSPERO, CDR4202344964.

The randomized controlled trials (RCTs) and Observational studies were pooled carefully based on title, abstract, full review text, tables, and figures for inclusion in the final analysis. While commentaries, case reports, letters to the editors, and corresponding articles were excluded. The duplicates were removed using The EndNote Reference Manager (Version X7.5; Clarivate Analytics, Philadelphia, Pennsylvania).

### Inclusion and exclusion criteria

We have included all clinical trials and observational studies in the English language assessing the efficacy of tafamidis in transthyretin cardiac amyloidosis. The selection criteria were based on (a) age >18 years and (b) patients were randomized to taking tafamidis 80 mg against the control group. All other study designs were excluded: (a) single-arm intervention, (b) age <18 years, and (c) primary intervention dose less than 80 mg.

### Statistical analysis and risk of bias assessment

The Review Manager Version 5.4 Cochrane Collaboration was used to analyze pooled data using random and fixed models to calculate the odds ratio (OR) and its 95% confidence interval (CI) to extract dichotomous outcomes. A fixed model is used for outcome-sharing similar effects with the intention to apply results for included studies only. Furthermore, a random effect model was used for outcomes to yield generalized results and conclusions for continuous outcomes and the hazard ratio (HR) to analyze the association of genetic variants of the ATTR-CM, ATTRwt, and ATTRvt. The results are demonstrated as forest plots. A *P*-value of 0.05 or less was considered significant for the outcome. The heterogeneity was assessed using Higgins *I* ^2^ statistics. A value of less than 50% was considered mild, greater than 50–75% as moderate, and greater than 75% was considered severe heterogeneity. No funnel plots were used as the total number of studies was less than 10. Cochrane Collaboration’s risk of bias 2.0 (ROB 2.0) tool to evaluate the quality of included RCTs using five domains (randomization, intended intervention, missing data, outcome measurement, and reported results) was carried out by two independent authors M.S. and S.R., later discussed to resolve any discrepancy.

## Results

### Study selection and characteristics

The PRISMA flow depicts the study selection process Figure A (Supplemental Digital Content 3, http://links.lww.com/MS9/A296). The initial search yielded 235 articles, of which we pooled two RCTs and one retrospective study, which matched our inclusion criteria to evaluate the efficacy of tafamidis when compared to the control group in patients of ATTR-CM. A total of 876 patients were included in this meta-analysis. The summary of the demographic characteristics of the included studies is demonstrated in Table 1. The ATTR-LTE is a long extension study that enrolled patients after completing the ATTR-ACT trial. Patients on placebo in the ATTR-ACT trial had been assigned to tafamidis 20 mg, which we included in our study as the control group, in comparison to the continuous tafamidis group, which showed excellent response.

**Table 1:** Baseline clinical characteristics of the included studies in this meta-analysis

Sr. No	Study, year	Registration	Type of study	Population	Intervention	Comparator
1	ATTR-ACT (2018)^5,6^	NCT01994889	Phase III Double-blind Randomized placebo-controlled trial (multicenter)	Age 18–90 years with ATTR-CM confirmed the presence of amyloid deposition by biopsy	Tafamidis (80 and 20 mg)	Placebo
2	ATTR-ACT LTE (2021)^7^	NCT02791230	Phase III Double-blind Randomized placebo-controlled trial (multicenter)	Patients in ATTR-ACT trial who were on tafamidis 80 mg and patients in the Placebo group. (Patients on 20 mg tafamidis were excluded)	Continuing tafamidis free acid 61 mg	Placebo to tafamidis
3	Ochi *et al*. (2022)^8^	–	Retrospective study	Patients with ATTR-CM (ATTRwt variant) confirmed by biopsy	Tafamidis 80 mg	Control group

### Quality assessment

The revised Cochrane tool for the risk of bias (ROB 2.0) has been used for quality assessment^9^. Overall, both RCTs ATTR-ACT and ATTR-ACT LTE were considered low risk for any publication bias for randomization quality, procedure, and analysis. The figure for risk of bias is available in the Supplementary Material (Supplemental Digital Content 4, http://links.lww.com/MS9/A297).

### Cardiovascular mortality

Results demonstrated that in patients with ATTR-CM, tafamidis showed a significant effect in the reduction of cardiovascular (CV) mortality in ATTR-ACT trial and Ochi *et al*. (OR 0.58; 95% CI: [0.41–0.83], *P*=0.003, *I* ^2^=87%; Fig. 1.1.1)^6,8^. To better characterize this outcome, a subgroup analysis was conducted for CV mortality due to heart failure (OR 0.89; 95% CI: [0.63–1.25], *P*=0.50, *I* ^2^=93%; Fig. 1.1.2).

**Figure 1:**
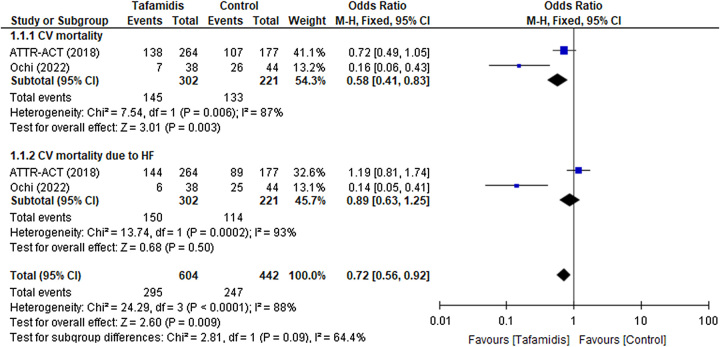
(1.1) Cardiovascular (CV) mortality amongst patients with ATTR-CM taking Tafamidis vs. Control intervention. (1.2) Subgroup CV mortality due to heart failure. ATTR-CM, transthyretin amyloid cardiomyopathy.

#### Cardiovascular mortality due to heart failure

Tafamidis showed a decrease in CV mortality due to heart failure in the study by Ochi *et al*., while in the ATTR-ACT trial, it did not exhibit any reduction^6,8^. However, according to the fixed effect model, the comprehensive results from the forest plot appeared to give a remarkable reduction in CV mortality with an overall effect estimate (OR 0.72; 95% CI: [0.56–0.92], *P*=0.009, *I* ^2^=88%; Fig. 1). Subsequently, the marked heterogeneity raises a concern which can be attributed due to (a) variable age groups, ATTR-ACT (≥18 to ≤90 years) and Ochi *et al*. (81±06 years). (b) Fewer study data and variable different analytical approaches in RCTs and observational studies. (c) The difference in the disease population, a study by Ochi *et al*. (2022) demonstrated the result of the Japanese population, whereas other studies include American, European, and Japanese populations.

### All-cause of mortality

A marked decrement was seen in all-cause of mortality when tafamidis was given in fixed doses of 80 and 20 mg in the ATTR-ACT trial and continuing 80 mg tafamidis in ATTR-ACT LTE trial (OR 0.52; 95% CI: [0.39–0.70], *P*≤0.0001, *I* ^2^²=0%; Fig. 2.1)^1,6,7^.

**Figure 2:**
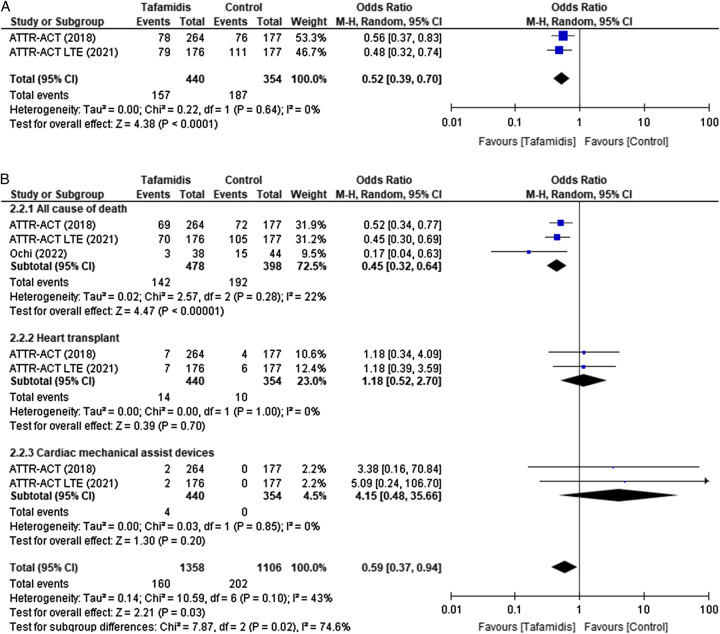
(2.1) All causes of mortality in patients with ATTR-CM taking Tafamidis vs. Control intervention. (2.2) Subgroup analysis of mortality. (2.2.1) All causes of death, (2.2.2) heart transplant, and (2.2.3) cardiac assist devices. ATTR-CM, transthyretin amyloid cardiomyopathy.

#### Death, patients with heart transplants, and cardiac assist devices

A subgroup analysis was carried out in which all causes of death, patients with a heart transplant, and cardiac mechanical assist devices were compared to see the effect of tafamidis on such patients. All three studies, that is, ATTR-ACT, ATTR-ACT LTE, and Ochi *et al*. manifested a decline in all-cause of death with tafamidis (OR 0.45; 95% CI: [0.32–0.64], *P*=<0.00001, *I* ^2^=22%; Fig. 2.2.1)^6–8^. Nevertheless, patients with heart transplant (OR 1.18; 95% CI: [0.52–2.70], *P*=0.70, *I* ^2^=0%; Fig. 2.2.2) and cardiac mechanical assist devices (OR 4.15; 95% CI: [0.48–35.66], *P*=0.20, *I* ^2^=0%; Fig. 2.2.3) portrayed no effect from tafamidis in both ATTR-ACT and ATTR-ACT LE trials. Even so, the overall effect estimate of subgroup analysis in the random effect model showed a significant decline in mortality (OR 0.59; 95% CI: [0.37–0.94], *P*=0.03, *I* ^2^=43%; Fig. 2.2) with moderate heterogeneity based on discrepancy in demographic factors or minimum study inclusion in our meta-analysis.

#### ATTRwt and ATTRvt variants

**Figure 3:**
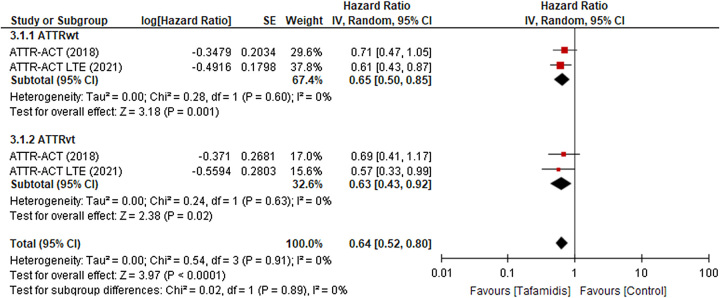
All causes of mortality in the two most common variants of ATTR-CM, ATTRwt, and ATTRvt. ATTR-CM, transthyretin amyloid cardiomyopathy.

On the other hand, patients with the two variants of ATTR, that is, ATTRwt and ATTRvt were administered tafamidis with randomized doses; the size of the effect was obtained in the form of the hazard ratio, patients with ATTRwt revealed abatement in all-cause of mortality (HR 0.65; 95% CI: [0.50–0.85], *P*=0.001, *I* ^2^=0%; Fig. 3.1.1) while patients with ATTRvt also gave the same outcome (HR 0.63; 95% CI: [0.43–0.92], *P*=0.02, *I* ^2^=0%; Fig. 3.1.2). In both the variants of ATTR, tafamidis manifested reduction in all-cause of mortality with total effect estimate (HR 0.64; 95% CI: [0.52–0.80], *P*≤0.0001, *I* ^2^=0%; Fig. 3)^5,7^.

## Discussion

To the best of our knowledge, this is the first double-arm meta-analysis assessing the efficacy of tafamidis on patients with ATTR-CM. The primary findings of our study indicate that tafamidis is superior to the control group in reducing the combination of CV mortality. Tafamidis also shows a significant decrease in all causes of mortality but does not significantly support heart transplants and cardiac mechanical assist devices. Anyhow, we observed a consistent overall benefit from tafamidis related to mortality across all subgroups. Patients with the two most common variants of ATTR, that is, ATTRwt and ATTRvt, evinced great reduction in all-cause of mortality when administered with tafamidis. This suggests that tafamidis is a safe and efficient drug to use in patients with ATTR-CM and can possess the potential to be a milestone in enhancing the conservative management of the patients.

The potential clinical implications of these findings are concomitant with the data in the literature. A systematic review by Singh *et al*. demonstrates the potential halting of disease progression by tafamidis causes a reduction in cardiovascular mortality and all causes of mortality^10^. The therapeutic effect of tafamidis can be described based on its mechanism of action, that is, tafamidis binds with selectivity, high affinity, and negative cooperativity (K D1–2 nM, K D2–200 nM) to transthyretin in human plasma. Tafamidis parades comparable potency against dissociation of heterotetramers of greater than 35 TTR mutants, including the two most common variants, and slows down the rate of tetramer dissociation at physiologic pH in a dose-dependent manner^11^. However, the type of adverse effects seen with tafamidis is similar to the control groups. Discontinuation of the drug leading to adverse effects is less common in patients who received tafamidis than in those who were in the control group, and dose reductions are uncommon and occur more often in the control group^1^. There was no pharmacotherapy for ATTR before tafamidis and OLT was the only treatment for patients with ATTR. These findings indicate that tafamidis is effective in patients with ATTR-CM and can be promising in reducing the progression of the disease.

### Limitations

We included subgroup analysis in this meta-analysis, which strengthens the outcomes and demonstrates the efficacy of the treatment in different genetic variants of disease and contrasting patient severity of the medical condition. Nevertheless, only three studies were included in this meta-analysis, which is a possible limitation and may create a risk of bias. Publication bias may be encountered in this following review as both RCTs included the same population, while the observational study included the Japanese population. Therefore, for the future direction, we encourage further conduction of the drug trial globally to support the evidence of the efficacy of tafamidis.

## Conclusion

Based on the results of our systematic review and meta-analysis, it is concluded that tafamidis decreases CV mortality and all-cause of mortality when given to patients diagnosed with ATTR-CM in all subgroups; however, patients with heart transplants and cardiac mechanical assist devices are not the best candidate for tafamidis. This meta-analysis suggests that tafamidis is a safe and efficient drug to use in patients with ATTR-CM and can possess the potential to be a milestone in enhancing the conservative management of the patients.

## Ethical approval

Ethics approval was not required for this review.

## Consent

Informed consent was not required for this review.

## Sources of funding

Not applicable.

## Author contribution

M.S.: conception, study design, data extraction, data analysis, writing, and reviewing-editing; S.R.: data extraction, writing, and reviewing-editing; M.W.: data extraction, writing, and reviewing-editing; M.S.: writing and reviewing-editing; R.R.: reviewing-editing; N.A.: reviewing-editing; S.O.: reviewing-editing; Md.A.H.: reviewing-editing.

## Conflicts of interest disclosure

There are no conflicts of interest.

## Research registration unique identifying number (UIN)

1. Name of the registry: PROSPERO.
2. Unique identifying number or registration ID: CRD42023449964.
3. Hyperlink to your specific registration (must be publicly accessible and will be checked): https://www.crd.york.ac.uk/prospero/#recordDetails.

## Guarantor

Shafi Rehman, e-mail: shafirehman@hotmail.com.

## Data availability statement

Data are available on request.

## Provenance and peer review

Commissioned, externally peer-reviewed.

## Supplementary Material

SUPPLEMENTARY MATERIAL
