# Supplementary material for: Efficacy of tafamidis in transthyretin amyloid cardiomyopathy: a systematic review and meta-analysis
Source: Ann Med Surg (Lond). 2023 Nov 7;86(1):433–8. doi: 10.1097/MS9.0000000000001482 (PMC10783421; doi:10.1097/MS9.0000000000001482)
Supplement: SUPPLEMENTARY MATERIAL [file ms9-86-433-s003.pdf]

Intention-to-treat	Unique ID	Study ID	Experimental	Comparator	Outcome	Weight	D1	D2	D3	D4	D5	Overall
	NA	Elliot et al.	Tafamidis	Control	NA	NA	+	+	+	+	!	+
	NA	Rapezzi et al.	Tafamidis	Placebo	NA	NA	+	+	+	+	!	+

+	Low risk
!	Some concerns
-	High risk

D1	Randomisation process
D2	Deviations from the intended interventions
D3	Missing outcome data
D4	Measurement of the outcome
D5	Selection of the reported result
